# Kill rate as a tool in efficiency evaluation of *Neoseiulus californicus* (Acari: Phytoseiidae) mass reared on factitious food

**DOI:** 10.1093/jisesa/iead061

**Published:** 2023-09-18

**Authors:** Sauro Simoni, Giovanni Burgio, Franca Tarchi, Silvia Guidi, Donatella Goggioli, Elena Gagnarli, Francesco Turillazzi, Alberto Lanzoni

**Affiliations:** Council for Agricultural Research and Economics, Research Centre for Plant Protection and Certification (CREA-DC), Florence, Italy; Department of Agricultural and FoodSciences, University of Bologna, Bologna, Italy; Council for Agricultural Research and Economics, Research Centre for Plant Protection and Certification (CREA-DC), Florence, Italy; Council for Agricultural Research and Economics, Research Centre for Plant Protection and Certification (CREA-DC), Florence, Italy; Council for Agricultural Research and Economics, Research Centre for Plant Protection and Certification (CREA-DC), Florence, Italy; Council for Agricultural Research and Economics, Research Centre for Plant Protection and Certification (CREA-DC), Florence, Italy; Council for Agricultural Research and Economics, Research Centre for Plant Protection and Certification (CREA-DC), Florence, Italy; Department of Agricultural and FoodSciences, University of Bologna, Bologna, Italy

**Keywords:** Neoseiulus californicus, Lepidoglyphus destructor, Glycyphagus domesticus, Quercus sp. pollen

## Abstract

The predatory mites of the Phytoseiidae family are crucial biological control agents widely utilized in biological pest management targeting phytophagous mites and insects. Key factors in these control strategies are that phytoseiids must be able to find their main target prey and to maintain high populations and efficacy. To reduce expenses and time-consuming production methods of mass rearing of phytoseiids, pollen and other factitious (i.e., non-natural/nontarget) hosts need to be present as an alternative food for predatory mite populations. The mass-rearing possibilities of these predators on alternative food sources, such as astigmatid mites (i.e., house and stored mites) and pollen, must be evaluated not only by the cost of rearing settings but on the basis of their efficiency maintenance in killing prey. The pest kill rate (*k*_*m*_) is the average daily lifetime killing of the pest by the natural enemy under consideration and can represent a useful indicator for phytoseiids efficacy to rank them as BCAs on the basis of their effective killing/predation on target prey. In this study, we evidenced that 2 astigmatid mites, *Glycyphagus domesticus* (De Geer) and *Lepidoglyphus destructor* (Schrank), and *Quercus ilex* L. pollen can be successfully adopted as substitute food sources for mass rearing of the phytoseiid *Neoseiulus californicus* (MgGregor); then, we determined that these populations of BCAs maintained a consistent *k*_*m*_ at new/first impact on the main target pest, *Tetranychus urticae* Koch.

## Introduction

Extensive research has been performed to evaluate BCAs, how to manage them, and to define guidelines/indications about screening and selection of the most efficient predators/parasitoids (Bale et al. 2008, van Lenteren 2012, Barratt et al. 2018, McEvoy 2018, Sentis et al. 2022). Many approaches have been evaluated, from typically empirical approaches, such as collecting and releasing any natural enemy attacking the pest (see “hit or miss approach”; DeBach 1964), to time-consuming research programs, including behavioral and ecological studies, and environmental risk assessments. The combination of several approaches can support the production/use of BCAs to optimize effective and sustainable management, for example under consideration of changing environmental conditions in the agroecosystems (Sentis et al. 2022).

Over the past decades, the intrinsic rate of increase, *r*_m_, (Birch 1948) has frequently been taken into account when assessing a predator’s potential or making comparisons between different predators (Southwood 1966, Sabelis 1985, van Lenteren 2010). Trials comparing the intrinsic rates of increase and predation capacities were conducted with regard to phytoseiids and their possible mass rearing, with the perspective to be largely sold (see Janssen and Sabelis 1992). The effectiveness of predatory mites is largely due to their ability to reproduce in large numbers on cheap, both alternative and factitious, food sources (Vangansbeke et al. 2023).

In addition to reaching high populations of phytoseiids, a major issue is the assessment of the maintenance of the predatory efficiency, as evidenced by kill rate on the target species, for example *in Tetranychus urticae* Koch control strategies (Norman 2023, Vangansbeke 2023). During the past years, more and more mass rearings of phytoseiids on alternative prey/food have been conducted to reduce costs and time-consuming methods. It is to be considered that studies are frequently connotated by different approaches: ecologists mainly study how populations are managed but, also how one organism (a natural enemy) is used to lower the population density of another organism (a pest): in this case, it can be difficult to find a good and efficacious BCA among the hundreds of pests’ natural enemies (Bielza et al. 2020, Popov and Belyakova 2022).

The finding that astigmatid mites could be raised as food source for predatory phytoseiid mites, allowing the low-cost and bulk production of predators, was a significant advance in rearing methods (Ramakers and van Lieburg 1982). Although production systems described in the literature are still relatively few (Bolckmans and van Houten 2006, Simoni et al. 2006, Midthassel et al. 2013), many species of predatory mites are currently mass-produced and utilized with astigmatid mites and pollen, to enhance commercial mass rearing and utilization of some species of phytoseiids (Messelink et al. 2014, Pappas et al. 2017, Eini et al. 2022). The usage of pollen and (extrafloral) nectar as food supplements has significantly advanced biological control programs, both generating large populations of phytoseiid and supporting the predator population in the wild when the prey is scarce or absent (Messelink et al. 2014, Delisle et al., 2015, Pappas et al. 2017, Pijnakker et al. 2020, Eini et al. 2022).

While the *r*_m_ is indicative of the demographic parameters of the phytoseiids in different situations (food, abiotic conditions), to quantify the quality control of the phytoseiids mites, an effective indication of the predatory potential of the species must be considered. Taking this into account, as suggested by Tommasini et al. (2004) and van Lenteren et al. (2019), the adoption of the pest kill rate is worth of consideration, as aggregate datum to connote the real efficiency of a phytoseiid species. The pest kill rate (*k*_*m*_) is the weighted daily average of the lifetime killing of the host or prey due to actions (predation, parasitism, and nonreproductive prey and host killing) of a natural enemy (Van Lenteren et al. 2021). The kill rate is particularly indicative regarding phytoseiids mass reared on factitious and/or alternative food sources and once again facing and killing their target prey.

A variety of astigmatid storage mite species as potential alternate prey for the phytoseiid *Neoseiulus californicus* (McGregor), a largely adopted and marketed BCA, was screened (Castagnoli et al. 2007): stored food mites were chosen because the house dust mite *Dermatophagoides farinae* Hughes, a distinct astigmatid species, proved to be an excellent food source for *N. californicus* (Castagnoli et al. 1999). However, *D. farinae* is less suitable for industrial mass production due to its significant role in human allergic reactions (Colloff 2009): to ensure a controlled increase in the predator population, a viable substitute prey should also be readily available, inexpensive, easy to mass-produce, and, at the same time, without risk of allergy. Recently, Nguyen et al. (2013) screened an astigmatid mite by confirming the attainment of high populations in mass-reared *N. californicus* fed with *Lepidoglyphus destructor* (Schrank) (see Castagnoli et al. 2007). *Glycyphagus domesticus* (De Geer), easily to be maintained, was also considered as an alternative food for mass rearing of some phytoseiid species (Vangansbeke et al. 2023).

Frequently, the *r*_*m*_ was adopted to characterize the efficiency of phytoseiid response, for example, to new food or environment. However, it is questionable to what extent, the intrinsic rate of increase is a reliable indicator of how effectively a predator may regulate an ecosystem because this rate only shows how quickly a predator population grows and not how many prey individuals it can kill (van Lenteren 2010). It is consequently advisable to consider pest kill rates of different phytoseiid species aiming at comparing their capacity to decrease pest populations. In this regard, the potential of *k*_*m*_ as indicator could be implemented by the acquisition of wider patterns of data and definition of “standards” and key elements to calculate pest kill rate.

In the present study, we assessed the pest kill rate of the generalist phytoseiid *N. californicus*, which shows traits of both type II specialist predatory mites and type III generalist predatory mites (Castagnoli and Simoni 2003). Fertility levels and kill rates of *N. californicus*, on the target prey *T. urticae*, the red-spotted spider mite, are calculated and compared for the phytoseiid reared for over 2 yr, from 3 different mass-rearing systems: phytoseiid mites reared on the astigmatid mites, *G. domesticus* and *L. destructor*, and on *Quercus* sp. pollen. In order to use, in practice, the pest kill rate to rank phytoseiids as BCAs, care was posed to define the correlation of pest kill rates with different conditions and for predators with different feeding histories. Furthermore, here the contribution to pest kill rates was exactly calculated by considering also the amount of prey killed by immature stages.

## Materials and Methods

### Stock Cultures of *L. destructor* and *G. domesticus*

*Lepidoglyphus destructor* (Ld) was isolated from stored grains, while *G. domesticus* (Gd) was isolated from stored food in domestic kitchens; both mites were first raised and then maintained in pure culture by feeding with the following diet: 1/3 of de-bittered dry brewer’s yeast, 2/3 of a weaning product for newborn rabbits, and 1 g of animal fat per 100 g of yeast and feedstuff (Castagnoli et al. 1989). In the institutional laboratory and facilities, these rearings were maintained more than 2 yr under controlled conditions (18–20 °C, 80–85% RH). To favor an increase in astigmatid populations and to prevent mite escape and maintain high and constant humidity level (>80%), each astigmatid mite species was raised for several generations in plastic beckers (5 cm diameter) that were embedded in larger plastic container with KOH saturated solution (Winston and Bates 1960).

### Pollen Acquisition to Mass Rear Phytoseiids

Holm oak pollen (Po) is collected annually, at the beginning of the flowering season, occurring in May, in country areas far from Florence urban area to prevent risk of deriving pollution. The aments are removed from trees and allowed to fall directly into open paper bags and sheets of packing paper in the laboratory and left 3–5 days at room temperature (16–19 °C), until they completely opened. Pollen grains are gathered in a large glass Petri dish after the ejected pollen and dried anthers were filtered through a 500 mµ mesh screen. After being dried in an oven at 37 °C for 48 h, the collected pollen grains are kept at 20 °C for long-term storage and at 4 °C in a refrigerator for short-term storage during tests, as the monthly food source for the mites to reduce the risk of damage from potential thermal shock (Sabbatini Peverieri et al. 2006, Kolokytha et al. 2011).

### Maintenance of Phytoseiids to be Introduced on Gd–Ld–Po Stock Cultures

*Neoseiulus californicus* was originally collected on wild strawberry and then maintained for at least 2 yr, on excised strawberry leaves highly infested with *T. urticae* and placed on wet cotton in Petri dishes (9 cm diameter). These colonies were maintained in climatic chambers at 25 ± 1 °C, 75 ± 5% RH, 16L:8D-h photoperiod.

### Experimentation Techniques

To start the mass rearing more than 100 motile forms of phytoseiids were introduced into each becker containing *L. destructor* or *G. domesticus* or placed on capsula Petri (15cm diameter) containing *Quercus* pollen. These mass rearing were maintained at the cited climatic conditions and checked, regarding their status, for more than 2 yr.

After 2 yr, single eggs coming from the 3 (Ld, Gd, Po) different mass rearings of *N. californicus* (Nc) were isolated on *T. urticae* (Tu) infested strawberry leaflets (cultivar Honeoye) located in a Petri dish filled with water-soaked cotton. On each leaf, by strips of damp cotton wool it was delimited a roughly 4 cm^2^ arena (2 × 2 cm). It was carefully considered to daily maintain the following *T. urticae* charge for each leaf area unit: 20 eggs, 15 immatures (proto-, deutonymphs), 15 adults. Daily, if necessary, the addition or removal of the different stages of *T. urticae* was performed. These *T. urticae* stage densities were adopted to guarantee, based on literature and data on functional and numerical responses for *N. californicus* on *T. urticae*, an ad libitum daily number of the different stages of tetranychids (Zhu et al. 2019). Daily, each leaf area was examined, and the phytoseiid specimen moved to a new standard prey-charged arena.

The following data were daily registered: Nc survival; Nc stage; Nc eggs laid; Tu eggs killed; Tu immatures killed; Tu adults killed. Fifteen replicates were performed for each of the 3 situations considered. Then, the parameters for calculating the kill rate were established: juvenile and adult mortality, egg-to-egg time, daily fecundity, and number of *T. urticae* killed/day for each stage.

As the aim of the study was focused on the number of Tu prey killed, by considering literature data on developmental time and biological traits of *N. californicus*, the life cycle of the phytoseiid was portioned in 3 periods: from day 1 to 9, by including likely the reaching of adult stage and preoviposition time; from day 10 to 29 to study a period close to generation time, at 25 ± 1 °C, 75 ± 5% RH, 16:8 L:D; from day 30 to the female death (Castagnoli and Simoni 2003, Canlas et al. 2006). The phytoseiids were obtained from the strawberry leaf arenas that had previously been infested by *T. urticae* females for prey adjustment and eventual replacement of hatched eggs or other stages to reach the above-mentioned densities. At day 8, to a first newly observed female, just emerged from immature stage, it was added a male to allow mating, and, at the end of mating, the male was removed; mating chance was replicated every 2 wk.

The parameters for calculating the kill rate were available: juvenile/adult mortality, egg-to-egg time, daily fecundity, number of *T. urticae* killed/day paying attention to the tetranychid stage killed. The natural mortality of prey was considered to be comparable across the experimental groups as our laboratory preliminary findings suggested that it was essentially negligible (>96.5% survival of immature stages) under the conditions examined.

### Data Analysis

The effect of the origin of *N. californicus* (Ld, Gd, Po) on number of *T. urticae* killed was evaluated over the 3 periods of life cycle of the phytoseiids (<9, 10–29, >30 days) regarding both cumulative and egg/immature/adult tetranychids killed. At the same, by considering the origin effect of phytoseiids, it was evaluated the cumulative oviposition of females and relative percentages of eggs laid during the periods since the first oviposition. A generalized linear model (MANOVA approach) was used to verify the effect of phytoseiid origin on data of tetranychids’ killed and *N. californicus* oviposition. The responses significantly affected by the origin factor were processed by Tukey post hoc comparison test. All percentage data were arcsine transformed to stabilize variance (Sokal and Rohlf 1995). All the cited procedures were carried on by SPSS Statistics for Windows (2011).

### Kill Rate Calculation

The intrinsic rate of increase (*r*_*m*_) (Carey 1993) was used as a framework to calculate the pest kill rate (*k*_*m*_) of *N. californicus* continuously reared on the 3 alternative foods and then allowed to prey on *T. urticae ad libitum*. In the formula indicated in Carey (1993), age-specific predation (*k*_*x*_) was substituted to age-specific fertility (*m*_*x*_), by considering all the development and adult stage of the phytoseiid (van Lenteren et al. 2019).


$$\begin{array}{l} \sum \left(e^{-(k_{m}x)}l_{x}k_{x}\right)=1 \end{array}$$

where *k*_m_ = ln *K*_0_/*T*_*k*_ is the net consumption rate, *K*_0_ = ∑_*x*_*l*_*x*_*k*_*x*_ the number of prey killed by a predator throughout a generation corrected by age-specific mortality, and *T*_k_ (predation time) is the mean period during a generation where the specimens actually prey (Tommasini et al. 2004). We used the Euler–Lotka equation (Lotka 1907, Lotka and Sharpe 1911) to calculate the value of pest kill rate: Σ[e−(*k*_*mx*_)*l*_*x*_*k*_*x*_] = 1. The value of *k*_*m*_ is obtained solving iteratively the Euler–Lotka equation. The predation time, *T*_*k*_, can be considered as the time required to a population to predate at a rate of *K*_0_: it can be estimated by calculating the mean predator ages for each age × weighted by the net number of prey consumed at that age


$$\begin{array}{l} (T_{k}=\;\sum_{x}l_{x}k_{x}x/\sum_{x}l_{x}k_{x}). \end{array}$$

The calculation of these parameters was performed by modifying the program written by Hulting et al. (1990) and relative computational algorithms. The program calculates intrinsic rate of increase and other parameters, by calculating also a jacknife estimate of the rate which can be used to determine the interval of uncertainty (see Meyer et al. 1986). The multiple comparison of kill rates among the 3 situations was performed by the Newmann–Keuls sequential test, according to Snedecor and Cochran (1980).

## Results

Altogether, the pool of 15 *N. californicus* females coming from each of the 3 different mass-rearing origins (*L. destructor*, *G. domesticus*, *Quercus* pollen) was able to kill more than 5,000 *T. urticae* forms: 5,198 *T. urticae* forms were killed from the phytoseiids previously reared on *L. destructor*, 5,364 from those previously reared *G. domesticus*, 5,047 from those previously reared on *Quercus* pollen, respectively. The length of the third period considered in recording data, from the day 30 to the death of females, lasted for 2 wk nearly: 13.2 days for *N. californicus* coming from Ld, 13.5 from Gd, 11.3 from Po, without any difference in durations (*F*_2,42_ = 1.449; *P* = 0.246). Throughout life period, a single female of *N. californicus*, when previously reared on *L. destructor*, killed cumulatively 115.62 ± 13.15 (mean ± SE) *T. urticae* forms, from *G. domesticus* mass rearing 119.20 ± 13.21 forms, from *Quercus* pollen 112.16 ± 13.91 forms, respectively.

It was registered a similar killing proportion of the different stages (egg, immature, adult) of *T. urticae* by *N. californicus* from the 3 rearing systems tested: the eggs represented 41% (range 39–46%) of the total prey killed, the immatures 39% (range 36–42%), the adults 20% (15–22%), respectively. The different origin of *N. californicus*, mass reared on *L. destructor* or *G. domesticus* or *Quercus* sp. pollen and once again facing/preying on *T. urticae*, did not affect the number of tetranychids killed by each phytoseiid female, neither cumulatively (*F*_2,132_ = 0.993; *P* = 0.379) nor daily (*F*_2,132_ = 0.028; *P* = 0.972).

Table 1 shows the number of all *T. urticae* forms killed per day by a female of *N. californicus*; the only difference in killing regarded the mass-rearing origin of the phytoseiid (*F*_2,42_ = 13.76; *P* < 0.001) during 0- to 9-day period. By considering the 3 different periods of phytoseiid life, for the 3 phytoseiid origins (Ld: *F*_2,42_ = 290; *P* < 0.001; Gd: *F*_2,42_ = 1,112; *P* = 0.001; Po: *F*_2,42_ = 760.2; *P* < 0.001), the highest number of *T. urticae* daily killed was always registered during the period from the 10th to the 29th day (Table 1). Regarding the killed prey registered during the first life period considered (0–9 days), it is to remark that more than 70% occurred during the preoviposition period, the 8th and 9th days: 73.02% (62.50–78.95% range) for *N. californicus* coming from *L. destructor* origin, 70.49% (57.89–80.00% range) from *G. domesticus*, 73.94% (63.64–100% range) from *Quercus* pollen origin, respectively.

**Table 1.** *Tetranychus urticae* (mean ± SD) daily killed by *N. californicus* coming from the three mass rearings in the 3 periods of phytoseiid life cycle: from egg to preoviposition time included (to day 9); time equivalent to the generation time (from day 10 to day 29); residual life time (from day 30 to female death)

Mass rearing of *N. californicus*	Cumulative daily *T. urticae* killed (all tetranychid forms/*N. californicus* female)		
	0–9 days	10–29 days	>30 days
*L. destructor*	2.59 ± 0.68 Aa	11.64 ± 1.25 Ba	8.29 ± 1.01 Ca
*G. domesticus*	2.73 ± 0.32 Aa	11.98 ± 0.55 Ba	8.38 ± 0.64 Ca
*Quercus* sp. pollen	1.84 ± 0.36 Ab	12.06 ± 1.01 Ba	8.70 ± 0.59 Ca

By row, different capital letters indicate significance among the prey killed by phytoseiids of different mass-rearing origins. By column, different lowercase letters indicate significance among the prey killed by phytoseiids during each of the 3 life cycle periods considered. MANOVA, *P* < 0.05, Tukey post hoc comparison test.

**Fig. 1.**
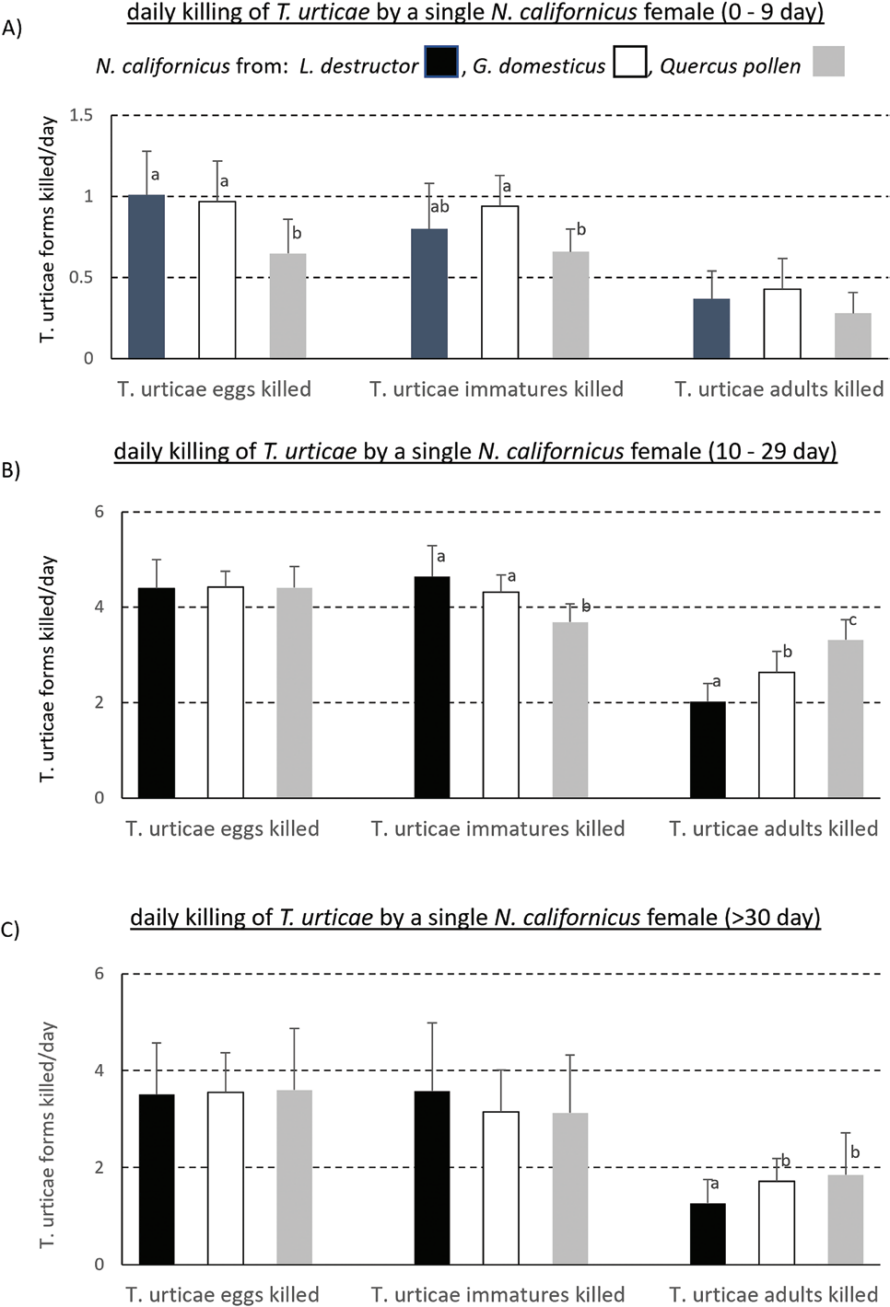
Daily killing of *T. urticae* (eggs, immatures, adults) by a single female of *N. californicus*, aged 0–9 days (a), aged 10–29 days (b), and aged over 30 days (c), coming from different rearings (*L. destructor*, *G. domesticus*, pollen).

Figure 1 shows the daily killing of different stages of *T. urticae* by *N. californicus* coming from the 3 origins. The lowest values were registered for pollen mass-reared *N. californicus*, 0–9 day aged, killing *T. urticae* eggs (*F*_2,42_ = 9.1; *P* < 0.01), *T. urticae* immatures (*F*_2,42_ = 6.41; *P* < 0.01) (Fig. 1a) and for the same originated phytoseiid, 10–29 days aged, killing *T. urticae* immatures (*F*_2,42_ = 14.26; *P* < 0.001) (Fig. 1b); lower values were for *L. destructor* mass-reared *N. californicus*, 10–29 day aged, killing *T. urticae* adults (*F*_2,42_ = 34.88; *P* < 0.001) (Fig. 1b) and for that, more than 30 day aged, killing *T. urticae* adults (*F*_2,42_ = 3.26; *P* < 0.01) (Fig. 1c).

A synthesis of oviposition data is reported in Table 2. Similar trends were observed in the 2 analyzed periods—10–29 days and >30 days—quite independently on the origin of the predator: *N. californicus* females, on the whole, laid from 60% to more than 70% eggs of their total eggs within the 30th day period.

**Table 2.** – Total and daily fecundity of *N. californicus* ovipositing females in the 2 life periods considered: over generation time calculated since the first egg laid (10-29d) and residual lifetime (>30d). By column, means followed by different letters are significantly different

	Total number of eggs laid/female/period		Daily number of eggs laid/female/period	
	10-29 day	>30 day	10-29 day	>30 day
*N. californicus f*rom				
*L. destructor*	47.47 ± 6.07a	13.48 ± 9.30b	2.50 ± 0.32a	0.71 ± 0.51b
*G. domesticus*	42.80 ± 7.34a	20.00 ± 9.69b	2.25 ± 0.49a	1.05 ± 0.20bc
*Quercus* sp. pollen	45.60 ± 3.80a	12.07 ± 3.39b	2.40 ± 0.39ac	0.64 ± 0.18b

(Manova, P<0.05, Tukey post hoc comparison test).

**Fig. 2.**
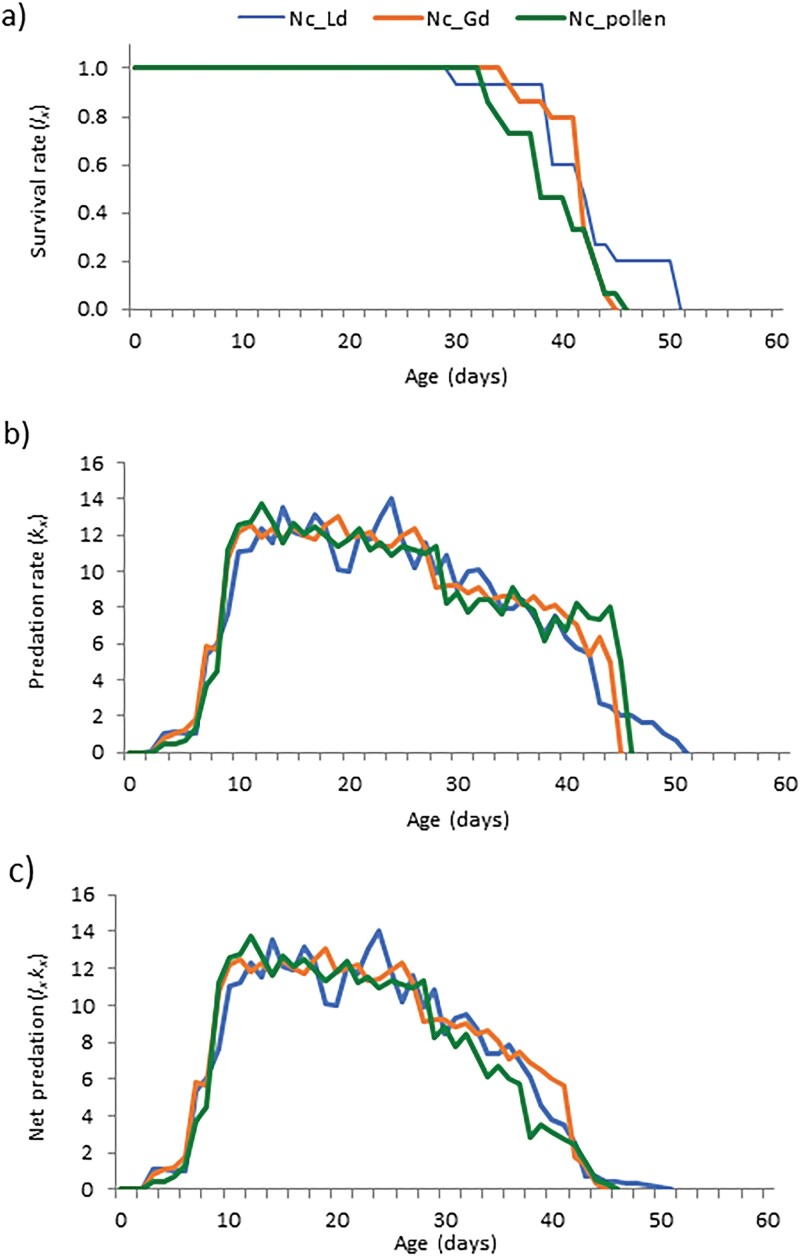
Rates of survival (a), predation (b), net predation (c) of *N. californicus* previously reared on 3 different foods (*L. destructor*, Nc_Ld; *G. domesticus*, Nc_Gd; *Quercus* sp. Pollen, Nc_pollen) at first preying impact on *T. urticae* at 25 °C and 75 ± 5% RH.

Figure 2 describes the age-specific survival (*l*_*x*_) (Fig. 2a), predation (*k*_*x*_) (Fig. 2b), and net predation (*l*_*x*_*k*_*x*_) (Fig. 2c) rates of *N. californicus*, previously reared and maintained on the 3 distinct types of food, at its new impact on *T. urticae* prey. In the 3 different rearing scenarios examined, the trends of *l*_*x*_ and *k*_*x*_ were comparable (Fig. 2a and b). The values of *k*_*m*_ were always high for *N. californicus* coming from the 3 different mass-rearing scenarios; the only difference was between the rate calculated for the phytoseiids reared on pollen in comparison with those previously reared on *G. domesticus.* Slightly lower values of *K*_0_ and *k*_*m*_ were found for *N. californicus* that had previously been fed with pollen (Table 3). Even not significantly, slightly better parameters were recorded for the phytoseiids previously mass reared on astigmatids in comparison with those reared on oak pollen (Table 3).

**Table 3.** Net consumption rate (***K***_***0***_), mean predation time (***T***_***k***_) and pest kill rate (km) of the predator *N. californicus*, previously mass-reared on three different alternative diet, at initial preying impact employing the red spotted spider mite as prey.

Parameters	Estimated killing on *T. urticae* for *N. californicus* previously reared on		
	*L. destructor*	*G. domesticus*	*Quercus* sp. pollen
*K*_*m*_ *(complete data estimate of rate)*	0.515	0.517	0.475
*Jacknife Estimate* *of K ± SE*	0.515 ± 0.015 ab	0.517 ± 0.007 a	0.475 ± 0.006 b
*Interval Estimate* *for K*	[0.483, 0.547]	[0.504, 0.531]	[0.463, 0.487]
*T*_*k*_ *(Mean Predation Time)*	11.36	11.38	12.25
*K*_*0*_ *(Net consumption* *Rate) ± SE*	347.13 ± 12.587	347.53 ± 5.768	336.47 ± 12.123

*Estimates followed by different letters are different (P<0.05) according to Newmann-Keuls sequential*

As a result of the parameters on tetranychid killing, all the *N. californicus* coming from the 3 different origins were able to double the whole predation within 2 days: *N. californicus* coming from *L. destructor* origin needs 1.35 days, from *G. domesticus* 1.34 days, from *Quercus* pollen 1.46 days, respectively.

## Discussion

These results suggest that mass rearing of *N. californicus* for several generations on different prey (*L. destructor*, *G. domesticus*) or pollen does not reduce its ability to kill the main target prey, in this case *T. urticae*, when facing the tetranychid for the first time.

Generally, literature reports higher *r*_*m*_ values for the tetranychids than for phytoseiids registered in similar conditions (Sabelis, 1985); in our study, the *k*_*m*_ values are higher than the *r*_*m*_ values for *T. urticae*, suggesting that the phytoseiid may kill more eggs, nymphs, and adults per day than the spider mites can produce.

Actually, when using *G. domesticus* as prey, results seem to provide a high phytoseiid population and high predation effectiveness, giving the phytoseiid species appropriate and effective possibilities to limit/control the target prey. In this study, there were evidenced consumption levels of different *T. urticae* stages similar to those registered by Canlas et al. (2006) on a Japanese strain of *N. californicus.*

Pest kill rate can provide better insights and effective information for estimating the potential of a natural enemy to control a pest (Van Lenteren et al., 2021). Consistently, in order to use the pest kill rate to rank phytoseiids as BCAs, some other issues should also be considered and integrated: establish the correlation of pest kill rate with intrinsic rate of increase, then compare those rates among different conditions and for predators with different feeding histories. However, caution must be taken in addition to the previous comparisons; the contribution in estimated values of the pest kill rates of the different developmental stages (immature stages, adult males, and females separately) should also be taken into account.

Although the adult female is the stage that most greatly contributes to determine the values of pest kill rates, other factors may be particularly pertinent for phytoseiid populations with varied stable age distributions and cannot be left out: for example, when a predator population is increasing, there is a conspicuous portion of immature stages and the consumption of these stages can importantly determine kill rate. In addition to the number of prey killed, it may be important to consider eventual difference in energetic budget assumed from prey stage consumed (eggs–immatures–adults), within the same prey species or different mite (or insect) species. Possible differences in prey exploitation level can also contribute to quantify the kill rate: some predators kill a large number of prey without consuming the entire body, while vice versa for others. These are all issues worthy of consideration and can corroborate the information on the duration of high kill rates. For example, can these rates be maintained over periods with rarefaction of prey? or with prey with particular age distribution (i.e., large portion of adults in the populations)? This information is not directly related to the increasing numerical growth, but can represent integrative information on the energetic budget gained in the more or less intensive exploitation of the prey and could be used in previsional phytoseiid release strategy.

Concerning the feeding of phytoseiids on immatures, there are variable data in literature. In the present study, the percentage of killed *T. urticae* immatures was very limited, sensibly below 5%. Gotoh et al. (2004) reported that larvae of 3 phytoseiid species (*Iphiseius degenerans*, *Metaseiulus occidentalis*, and *N. californicus*) started feeding soon after they hatched. Like what has been observed with other predatory mites, Gilstrap and Friese (1985) evidenced that the larvae of *A. californicus* appeared to be feeding. On the contrary, the larvae of other phytoseiid mites, including *Phytoseiulus macropilis* and *P. persimilis*, are reported as nonfeeding (Takafuji and Chant 1976).

To explain the presence of nonfeeding and feeding larvae/nymphs among the phytoseiid species, Chittenden and Saito (2001) report on relationships between prey preference, manner of oviposition, and larval feeding behavior of some phytoseiids. Obligatorily feeding larvae prefer prey species sparsely distributed and lay their eggs in a scattered fashion; nonfeeding and facultatively feeding larvae prefer prey species with a high aggregation and lay their eggs in a clumped fashion. Chittenden and Saito (2001) hypothesize that nonfeeding larval behavior may be adaptation to avoid sibling cannibalism, which occurs when eggs are closely oviposited, and this could also be important in pest control contexts.

Regarding factitious food used to mass rear phytoseiids, many species of astigmatid mites have been evaluated for use in the mass production, such as *Acarus farris* (Oudemans) (Ramakers and van Lieburg 1982), *Carpoglyphus lactis* (L.) (Bolckmans and van Houten 2006, Nguyen et al. 2013), *Suidasia medanenesis* (Oudemans) (Midthassel et al. 2013), *Lepidoglyphus destructor* (Schrank) (Simoni et al. 2006), and *Tyrophagus putrescentiae* (Schrank) (Riahi et al. 2017). However, little is known about the keeping in performance of different phytoseiids reared on different astigmatid mite species when they are released in field and/or greenhouse environment on target pest. Due to different environmental circumstances in crops compared to mass-rearing systems, prey mites that are suitable for mass rearing may not necessarily be suitable for application in the crop. Furthermore, it is relevant to determine whether astigmatid mite populations may pose a threat to human health due to their link to allergies and potential damaging of young and fragile plant tissues (Johansson et al. 1994, Hubert et al. 2018). To face possible risk of cross-contamination, freeze-killing the mites, in particular astigmatids as rich source of allergens, before treatment, may reduce the likelihood of allergies but the maintaining of performance should be carefully evaluated: according to a laboratory analysis of the astigmatid mite *T. putrescentiae* by Pirayeshfar et al. (2020), frozen stages could support *A. swirskii* development and oviposition in rearing but not in its action on greenhouse plants.

Generally, studies are primarily focusing on cost-effective and efficient factitious prey for mass production of phytoseiids, assessing predator quality when compared with natural prey. Nevertheless, it is highly expected to review the species of storage mites also in light of an increase in sensitization rates in different countries and occupations, and co-sensitization rates to house dust mites in EU countries (Cuevas et al. 2022). Attention must be paid to people particularly vulnerable to storage mite exposure because of their jobs: frequently factitious prey/food can generate critical allergic issues in farmers and agricultural, grain, and food workers.

In this study, the reaching of high populations of *N. californicus* on astigmatid mites and pollen is confirmed (see Tung et al. 2022). In addition, high performances in *T. urticae* killing capacity of *N. californicus*, newly facing the target prey, are maintained, particularly when the phytoseiid is coming from the astigmatids’ mass rearings.

When the phytoseiid species is involved in augmentative biocontrol systems, it is very important to maintain predatory efficiency. Although the predation of nymphs should also be taken into account for *k*_*m*_ calculation, along with the contribution of males, to maximize the predictive value of the pest kill rate as estimator of mass-reared predatory mite biocontrol capability, the *k*_*m*_ seems to be a suitable parameter (see van Lenteren et al. 2019).
